# Systemic differences in serum metabolome: a cross sectional comparison of women with localised and widespread pain and controls

**DOI:** 10.1038/srep15925

**Published:** 2015-11-02

**Authors:** J. Hadrévi, M. Björklund, E. Kosek, S. Hällgren, H. Antti, M. Fahlström, F. Hellström

**Affiliations:** 1 Department of Community Medicine and Rehabilitation, Sports Medicine Unit, Umeå University, SE 90187, Umeå, Sweden; 2 Department of Occupational and Public Health Sciences, Centre for Musculoskeletal Research, University of Gävle, SE 907 13, Umeå, Sweden; 3 Department of Community Medicine and Rehabilitation, Physiotherapy, Umeå University, SE 901 87, Umeå, Sweden; 4 Department of Clinical Neuroscience, Karolinska Institutet, SE 171 77, Stockholm, Sweden; 5 Department of Clinical Sciences, Professional Development, Umeå University, SE 901 87 Umeå, Sweden; 6 Department of Chemistry, Faculty of Science and Technology, Umeå University, SE 901 85 Umeå, Sweden

## Abstract

Chronic musculoskeletal pain exists either as localised to a single region or as widespread to multiple sites in several quadrants of the body. Prospective studies indicate that widespread pain could act as a far end of a continuum of musculoskeletal pain that started with chronic localised pain. The mechanism by which the transition from localised pain to widespread occurs is not clear, although many studies suggest it to be an altered metabolism. In this study, systemic metabolic differences between women with chronic localised neck-shoulder pain (NP), women with chronic widespread pain (CWP) and women who were healthy (CON) were assessed. Blood samples were analysed taking a metabolomics approach using gas chromatography mass spectrometry (GC-MS) and orthogonal partial least square discriminant analysis (OPLS-DA). The metabolomics analysis showed a clear systematic difference in the metabolic profiles between the subjects with NP and the CON but only a weak systematic difference between the subjects with CWP and the CON. This most likely reflects a difference in the portion of the metabolome influenced by the two pain conditions. In the NP group, the overall metabolic profile suggests that processes related to energy utilisation and lipid metabolism could be central aspects of mechanisms maintaining disorder.

Chronic musculoskeletal pain is frequent in the general population of western countries especially among women^1^,^2^ and one of the most common reasons for sick-leave. The pain may be located in single regions defined by individual localised structures (such as muscles and joints) or by areas like the neck and shoulders or lower back. Localised pain may be both specific and nonspecific, i.e. the presence of specific diagnosis or not. Pain can also be more widespread, i.e. generalised to multiple sites in several body quadrants. One of the most used definitions of chronic widespread pain (CWP) is the definition used in the American College of Rheumatology (ACR) criteria for fibromyalgia (FM), that includes pain in all the body quadrants, pain in the axial spine and a pain duration of at least 3 mon^3^,^4^. FM is a special form of CWP that also includes a finding of 11 or more trigger points out of 18, i.e. the ACR-criteria for FM^4^. The prevalence of CWP/FM^2^ and localised neck pain^5^ is higher among women than men. In addition, women show a higher risk of developing CWP/FM^6^ or localised neck pain^1^.

Localised pain and CWP have many symptoms in common, such as increased tenderness in the affected muscles^7^,^8^,^9^, exacerbation of pain during exercise^7^,^9^ and reduced muscle blood flow during static contractions^10^,^11^. However, patients with CWP/FM and those with localised pain differ regarding pain processing. A dysfunction of endogenous pain inhibition has been reported in CWP/FM^12^,^13^ and shown to be related to structural and functional cerebral abnormalities^14^,^15^, whereas a normal endogenous pain inhibition has been seen in patients with localised pain^8^. Furthermore, while a dysfunction of exercise-induced hypoalgesia (EIH) is seen in CWP as well as in patients with localised pain during contraction of painful muscle, normal EIH is seen in patients with localised pain during contraction of non-painful muscles^16^. These findings in combination with prospective studies indicating that localised pain is the major risk factor for developing CWP^17^ have led to the view of FM/CWP as being the far end of a continuum of chronic musculoskeletal pain states^18^,^19^.

It is generally believed that while the symptoms in patients or subjects with localised pain results from peripheral mechanisms, the pain in CWP/FM is more an effect of central pain amplification^18^,^19^. The pain, however, still relies on a complex interaction between peripheral and central mechanisms^20^. The mechanism by which pain makes the transition from being localised to CWP is not clear, although many studies regarding the genesis and maintenance of these pain conditions suggest an altered metabolism to be of importance^21^,^22^. Abnormalities in metabolism have been reported from muscles of patients suffering from both localised pain and CWP. Significant differences have been detected of metabolites such as glutamate, pyruvate and lactate^23^,^9^ as well as adenosine triphosphate (ATP) and poly creatine kinase (PCR)^24^.These findings indicate that changes in the systemic peripheral chemical environment may also have an impact on central mechanisms^25^. However, comprehensive overviews of the metabolic processes in localised pain and CWP are lacking.

This lack could be rectified by applying screening techniques such as metabolomics, which enable detection of a large number of small weight molecules, mirroring the metabolic processes and hence providing a snap-shot of the active overall biochemical processes. Recent metabolomics and proteomics investigations that compared myalgic trapezius muscle to healthy trapezius muscle indicate a difference in muscle metabolism between them; the investigations were reviewed by Hadrevi 2014^26^. In a metabolomics study,^22^ Hadrevi and co-workers analysed muscle interstitial fluid in myalgic and healthy trapezius muscle and provided a systematic overview of the local intra-muscular processes in myalgic muscle; they mainly presented differences in metabolic substances connected to general metabolism, pain and muscle turnover. Using metabolomics to analyse blood samples enables a systematic overview of the ongoing systemic processes, providing the possibility of comparing the previously detected local changes in muscle interstitium^22^ and possible systemic processes. This type of screening can also be used to detect previously unexplored metabolites in the context of local and widespread pain.

The aim of this study was to explore, by using metabolomics, the metabolite content and profile in blood serum samples from women with localised nonspecific neck-shoulder pain (NP) and others with CWP, and a group of controls who were healthy women (CON). The hypotheses are that in a comparison the group with localised pain and the group with generalised pain will exhibit different metabolite content and profiles from each other and from the healthy group, indicating different metabolic processes in the three groups. To this end we analysed serum blood samples from 30 subjects with NP, 16 with CWP and 39 CON, taking a metabolomics approach using gas chromatography mass spectrometry (GC-MS) and orthogonal partial least square discriminant analysis (OPLS-DA). The detection of potential systemic differences between these conditions will introduce new possible biomarkers enabling further assessment of the mechanisms of localised and chronic widespread pain.

## Results

### Subject characteristics

Thirty-nine healthy controls, 30 subjects with NP and 16 subjects with CWP, all female, were included in the study. There was no significant difference between groups when considering subject characteristics such as age, weight, body mass index (BMI), pain ratings at the time of blood sampling (NP and CWP groups only) and nicotine use (Table 1). Pain ratings that involved evaluating pain perception the previous week was significantly different (Mann-Whitney U test, p = 0.008) between NP and CWP as was pain duration (Mann-Whitney U test, p = 0.001), with a higher rating and longer duration for the CWP group (Table 1). Self-reported leisure physical activity was significantly different (Fischer exact, p = 0.001) between the CWP group and the CON, with a higher physical activity in the CON. However, no significant differences were detected between NP and CON (Fischer exact, p = 0.162) or between the NP and CWP groups (Fischer exact, p = 0.147) concerning self-reported leisure physical activity. No significant differences were detected between NP and CON or CWP and CON concerning self-reported frequency of intake of phytosterol rich food (Fischer exact, p = 0.411) 8 hours before blood samples were taken. Based on self-reports, all participants eat a varied diet including meat and fish and the composition of the food eaten within 8 hours prior to blood sampling contained fat, proteins and carbohydrates.

### Systematic differences between groups

**Figure 1:**
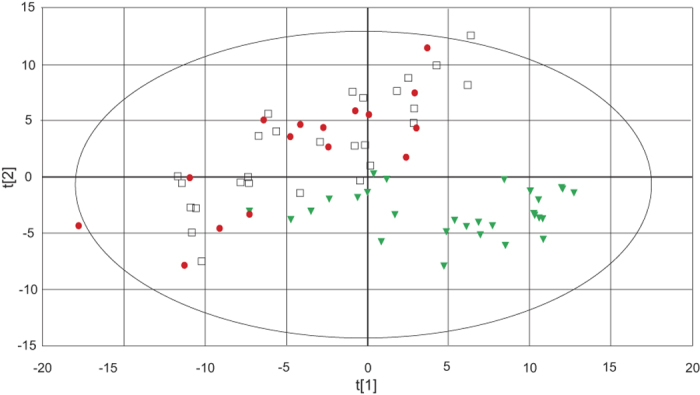
Principal component analysis (PCA). Score plot of component 1 (t[1]) and component 2 (2[t]). CWP patients (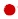), NP (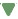) and CON (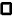). The score plot shows the internal correlation structure in the metabolite data. Three significant cross-validated principal components were used (R_2_X = 0.398; Q^2^ = 0.288). The ellipse shows 95% confidence interval using Hotelling T^2^ statistics.

In this study serum blood samples from 30 subjects with NP, 16 with CWP and 39 CON were analysed using taking a metabolomics approach, using GC-MS followed by multivariate data analysis by means of principal components analysis (PCA) and (OPLS-DA. In the GC-MS analysis 244 metabolites were detected and 110 of those were identified. All 244 metabolites were included in the multivariate analysis. An initial unsupervised PCA, revealed a clear separation of metabolites between NP from the CWP and CON groups (Fig. 1).

**Figure 2:**
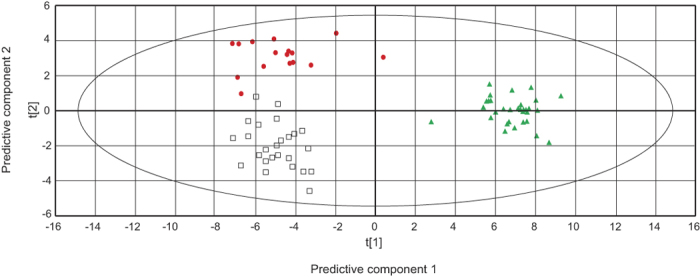
OPLS-DA score plot for the overview model including three groups. The model consists of two predictive component (p1,p2) shown in the figure and three orthogonal components (not shown). t[1] = scores for predictive component 1, t [2] = scores for predictive component 2. The model explains 41.9% of the variation in x-space and 51.1% of the variation in y-space. Explained variation of each components: R^2^X_p1_ = 0.145; R^2^X_p2_ = 0.02; R^2^X_o_ = 0.274. Goodness of prediction Q^2^_cum_ = 0.461. CWP (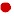), NP (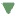) and CON (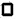). The ellipse shows the 95% confidence interval using Hotelling T^2^ statistics.

By applying supervised OPLS-DA, all three groups were separated in a model consisting of two predictive components (Fig. 2) and three orthogonal components (data not shown). To further elucidate the difference between CON–NP and between CON–CWP and to identify variables contributing to the differences, two separate OPLS-DA models were constructed, showing different metabolite profiles between CON and NP (R^2^X_p_ = 0.151; R^2^X_o_ = 0.285; Q^2^ = 0.928) and between CON and CWP (R^2^X_p_ = 0.03; R^2^X_o_ = 0.289; Q^2^ = 0.231).

### Identified metabolites

**Figure 3:**
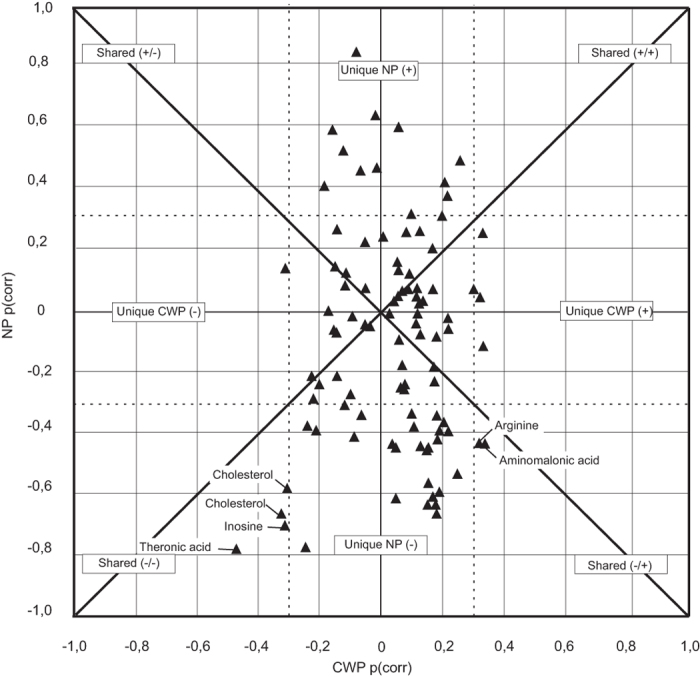
SUS-plot showing the shared and unique correlation structures between the CON–NP and CON–CWP OPLS-DA models. Dashed lines represents positive and negative correlation coefficients of 0.3.

Identified metabolites characterising NP in the CON–NP model were mainly fatty acids and metabolites related to carbohydrate and energy metabolism (Table 2). Table 3 shows the identified metabolites related to the CON in the CON–NP model, mainly amino acids and cholesterol but also glycerol-3-phosphate, α-tocopherol (vitamin E with anti-oxidative properties) and threonic acid (vitamin C metabolite). The CON–CWP model shows a very low explained variation (R^2^X_p_ = 0.03), thus a very small proportion of the metabolome contributes to the separation. This is also illustrated by the number of significant different metabolites between the CON and group with CWP as compared with the CON and group with NP. Identified metabolites in the CON–CWP model are presented in Table 4 and 5. In the SUS-plot, unique correlation structures in the metabolome that NP and CWP share are shown (Fig. 3). The plot shows a strong dominance of metabolites unique to the separation of CON vs NP. Only cholesterol, threonic acid and inosine show a lower abundance in both NP and CWP while arginine and aminomalonic acid show increased abundance in CWP and a lower in NP.

## Discussion

With this exploratory screening study we have detected systemic differences in metabolite abundance between three groups, one with NP, one with CWP and the CON. Using multivariate analysis methods show the CON and NP groups have the main systematic difference (Fig. 2). Although, cholesterol, inosine and threonic acid showed changes with a similar correlation structure in both NP and CWP and arginine and aminomalonic acid showed the opposite correlation structure (Fig. 3). The systematic difference between the CON and CWP and the CON and NP groups (Fig. 2) reflects the proportions of the systemic metabolome contributing to the respective group separations. The difference in explained variance of the predictive component between the CON–NP (R^2^X_p_ = 0.151) model and the CON–CWP (R^2^X_p_ = 0.03) indicates a proportionally larger systemic difference in metabolism during local chronic muscle pain than during widespread chronic pain. This may be due to adaptive processes changing the principle driving force of the pain sensation. It is generally believed that while the symptoms in patients or subjects with localised pain (NP in this study) result from peripheral mechanisms, the pain in CWP or FM groups is more an effect of central pain amplification^18^,^27^. In fact, CWP/FM is often regarded as being at far end of a continuum of musculoskeletal pain; this view is supported by the longer pain duration and higher self-rated pain intensities found in this study. Localised pain precedes CWP in the majority of individuals^28^ and is considered as a risk factor for development of CWP and FM^17^,^29^. CWP and FM are considered as resulting from a complex interaction between peripheral and central mechanisms, and it is believed that nociceptive input from muscles can initiate changes in cerebral pain processing^14^,^30^. However, there is evidence that both functional and structural cerebral abnormalities increase with increased duration of pain, suggesting that cerebral aberrations in pain processing become successively more important^15^.

In the present study, cholesterol, inosine and threonic acid showed changes with a similar correlation structure in both the NP and CWP groups (Fig. 3). In the univariate analysis, cholesterols and threonic acid showed a significantly lower abundance when comparing both healthy controls to CWP (Table 5) and NP and inosine only to NP (Table 3). Arginine and aminomalonic acid displayed opposite correlation structures with a higher abundance in CWP. However, neither was significant in the CON–CWP univariate analyses. Due to the lower explained variance in the CON–CWP model and the overall weak significance in the univariate analysis, the results from the CON–CWP must be considered with caution. Taking this into account, the following discussion on the possible interaction between metabolites is mainly based on the results from the CON–NP model.

Several amino acids, cysteine, serine, asparagine, arginine, orthinine, tyrosine and taurine, showed an increased abundance in the CON group as compared to the NP group and only valine, an increased abundance in the NP group. The functional relevance and cause of these changes in amino acids is difficult to fully deduce. However, cysteine with its thiol side chain has important antioxidant properties and together with glutamic acid (increased in the CON group) and glycine are the constituents of the antioxidant glutathione. Glutathione is oxidised to glutathione disulfide and reduced back using nicotinamide-adenine-dinucleotidephosphate (NADPH). Arginine and orthinine are both involved in the NADPH-dependent synthesis of nitric oxide. The role of nitric oxide in pain conditions is not clear with both nociceptive and analgesic effects peripherally and centrally (for review, see^31^). The main source of NADPH is the pentose phosphate pathway and the only metabolite identified in that pathway, erythrose-4-phosphate, showed an increased abundance in the CON group as compared to the NP group. In addition, α−tocopherol and threonic acid a metabolite of the antioxidant vitamin C, displayed an increased abundance in CON compared to NP. The results from this exploratory study suggest that further studies could to be directed toward investigating the role of oxidative stress in subjects with localised muscle pain.

One prominent difference between NP and CON is the lower abundance of cholesterol in the NP group. This difference between groups could be due to several mechanisms, including difference in intestinal absorption efficiency, biliary excretion and or synthesis of cholesterol though the mevalonate pathway.

In contrast to the present study, Ozgocmen *et al.* (2000) showed, in a study on 32 patients with myofascial pain syndrome (MPS) and 30 patients with FM syndrome, an increased total serum cholesterol levels in the MPS group compared to the age-matched healthy controls but no difference in comparison to the FM group^32^. In the study by Ozgocmen, a small but significant difference in body weight between the groups was observed^32^ in contrast to the present study where no differences in BMI or weight were observed between the groups (Table 1). Obesity or high BMI plays a role in upper extremity musculoskeletal disorders especially related to tendon disorders^33^. Several studies have related an increased serum level of cholesterol to work-related tendon disorders, for example in frozen shoulder^34^ and rotator cuff tear^35^. It is unclear if work-related tendon disorders were excluded in the study by Ozgocmen *et al.* (2000)^32^. In the current material the localised pain group consisted of subjects with nonspecific neck muscle pain, thus subjects with specific diagnosis like shoulder tendinitis were not included^36^. However, it must be emphasised that the mechanisms by which cholesterol could be lower are not clear in subjects with muscle pain.

In a speculation, a reduced cholesterol level in NP may arise from a reduced cholesterol synthesis by HMG-CoA reductase in the mevalonate pathway. A change in the mevalonate pathway reducing cholesterol synthesis, would also predict a reduction of other substances depending on the same pathway^37^, like testosterone and ubiquinone (coenzyme Q_10_). Ubiquinone is an antioxidant and essential component of the oxidative phosphorylation in the mitochondrial inner membrane. Lowered testosterone levels have been found in women suffering from clinically verified neck or shoulder pain disorders^38^. To the authors knowledge no study have investigate the levels of ubiquinone (coenzyme Q_10_) in people suffering from neck pain.

The methodology used did not enable analysis of ubiquinone, although there was an increased abundance of succinate in NP (Table 2). Accumulation of succinate occurs if the function of the redox process (i.e. transfer of electrons) in complex II is limited^39^ or if the activity or expression of succinate dehydrogenase is down-regulated^40^. In short, ubiquinone accepts electrons transferred from succinate by succinate dehydrogenase (complex II). In this process, succinate is oxidised to fumarate and ubiquinone is reduced to ubiquinol. Ubiquinol then delivers the electrons to complex III in the oxidative phosphorylation and is subsequently reoxidised to ubiquinone. A reduced production of ubiquinone (coenzyme Q_10_) could impair oxidative phosphorylation. Impaired oxidative phosphorylation has been proposed as one mechanism behind the adverse myalgia reported for cholesterol lowering pharmaceutical such as statins^41^.

The NP group showed an increased abundance in saturated fatty acids, myristic acid, nonanoic acid, palmitic acid, as well as in unsaturated fatty acids, gondoic acid, linoleic acid, oleic acid and arachidonic acid (Table 2). The saturated fatty acids myristic and palmatic acids were also evident in the muscle interstitium of the myalgic muscle^22^. The increased abundance of fatty acids in the NP group may be considered in relation to increased abundance of carbohydrate metabolites in the NP group (Table 2). The increased abundance of carbohydrate metabolites coincides with previously presented results of a reduction in glucose uptake in muscles in persons suffering NP compared to the muscles healthy controls^42^. In general, excess carbohydrate is converted to fatty acids, initially palmitic acid, mostly in the liver. A metabolite in the intersection of the glycolysis, fatty acid metabolism and oxidative phosphorylation is glycerol-3-phosphate (G3P), which showed a higher abundance in the CON group. Also Adenosine-5-monophosphate (AMP) was more abundant in the NP-group according to the CON-NP OPLS-DA model and the univariate statistics (Table 2). However, the implications of systemic differences of G3P and AMP for muscle pain are unclear.

Several unsaturated and saturated fatty acids are also precursor for synthesis of bioactive fatty acid amides related to inflammation and pain like N-acylethanolamines, including endocannabinoids^43^. Intra-muscular levels of N-acylethanolamines have been shown to correlate with pain intensity and sensitivity in women both with CWP and NP^44^.

In the present study, there was an increased abundance of taurine in the CON. Taurine, a sulphur-containing b-amino acid, is the most abundant amino acid in the body, with high concentrations in skeletal and heart muscle. The main source of taurine in humans is from diet, with endogenous synthesis from cysteine and methionine in presence of vitamin B_6_ being relatively small^45^. Taurine is involved in stress responses integrity of cell membranes and protein stabilisation (for review, see^46^) and interacts with the glutamate and GABA glucinergic system. Knock-out mice with taurine deficiency have been shown to have a reduced sensitivity to nociceptive chemical stimuli^47^, showing an effect of the peripheral nervous system. In this study, the NP group had less taurine in the blood stream compared to the healthy group and the CWP group, implying a potential correlation to pain perception.

The present study has a cross sectional design with independent groups thus no causal relationship between metabolites and muscle pain can be concluded. Also, when using a cross sectional design relevant confounding factors must be considered. Physical activity is one potential confounder in metabolomics analyses and a factor known to differ between subjects with different pain conditions and healthy subjects^48^,^49^. Physical activity has an impact on the general metabolism, increasing insulin sensitivity due to increased energy utilisation, hence altering the metabolite content in the blood stream^50^. In the present study, self-reported leisure physical activity did not differ significantly between the NP and CON group, thus the impact of different physical activity levels on the systemic differences in metabolic profiles may be considered weak.

An additional confounding factor could be the nutritional status of the participants. Dietary intake of phytosterols, like campesterol and β–sitosterol, competitively reduces the cholesterol intestinal absorption efficiency and could potentially account for the observed difference in cholesterol. In the present study, campesterol but not β–sitosterol showed a significant difference between the NP and CON groups (Table 2). This could indicate a difference in nutritional status between these groups. However, the significance of the groups difference in campesterol should be interpreted with caution: campesterol measured in plasma is not a suitable marker for dietary phytosterol uptake since the uptake mechanism is highly complex and not only dependent on intestinal concentrations^51^. Observations from diet intervention studies also suggest a large intra individual variation in phytosterol uptake^52^. A simple content analysis on the self-reported food intake 8 hour before blood sampling did not indicated any differences between groups, however this analysis does not preclude more long term difference in nutrition status. Also, all participants reported eating a varied diet including meat and fish. However, albeit steps were taken to minimise the potential impact of nutritional status and reasons for diverging nutrition between groups may be hard to conceive, differences between groups cannot be ruled out. Finally, the NP and CON groups were recruited from the same work places thus reducing the potential impact of uncontrolled workplace factors on the results.

The participating subjects were allowed to use paracetamol-based analgesics because their potential impact on metabolism is considered to be weak and the participants reported the amount taken during 3 day prior to blood sampling. Further, the suggested mechanism of paracetamol concerns more central pain mechanisms in combination with a weak anti-inflammatory effect^53^.

The present exploratory study explains the systematic differences in the systemic metabolic profiles of patients with two pain disorders, with localised nonspecific neck-shoulder pain (NP) and chronic widespread pain (CWP), and of healthy controls (CON). The results reflected differences in a portion of the systemic metabolome influenced by the two pain conditions, with a more pronounced involvement in those with NP. Several metabolites differed in abundance between NP and CON, indicating an overall difference in metabolic profile related to lipid metabolism and interrupted energy utilization. Longitudinal studies are needed in order to enable a more profound understanding of the mechanisms behind the development and maintenance of these pain conditions.

## Methods

### Subjects

Subjects were consecutively recruited into three groups: healthy controls (n = 40), subjects with localised nonspecific NP (n = 32) and subjects with CWP (n = 16). CON and NP subjects were recruited from a randomised controlled trial^36^ (Current Controlled Trials registration ISRCTN49348025).The subjects with CWP were recruited through contact with the local fibromyalgia patients’ organisation and advertising in local newspapers. The NP and CON groups were recruited from the same work places mainly the local university, local hospital and local municipality. Two subjects with localised nonspecific neck pain and one control were excluded from the data analysis because in a preliminary PCA they were found to be strong outliers (see method). One subject with NP was excluded because the blood sample showed haemolysis. The background subject characteristics are described in Table 1. Two questions^54^ were used to control for the level of physical activity at leisure time.

### Inclusion and exclusion criteria

Inclusion criteria for all groups were female and age between 20–65 years. For NP, the group inclusion criteria were nonspecific neck-shoulder pain indicated as a dominant pain area in a pain drawing, more than “no disability” but less than “complete disability” according to the Neck Disability Index (NDI), and self-reported impaired capacity on the quality or quantity to work done in the preceding month. More details on the inclusion and exclusion criteria for the NP group and the healthy control group have been described elsewhere^36^. Specific inclusion criteria for the CWP group were: diagnosed with CWP at time of investigation, i.e. following the ACR-criteria for FM^4^ but with no requirement of at least 11/18 trigger points.

The subjects with the following diagnosis were excluded from all groups: rheumatoid arthritis, systemic lupus erythematosus, Bechterew’s disease, multiple sclerosis, epilepsy or Parkinson’s disease, type 1 diabetes, cardiovascular disease, and endocrine diseases. Also, subjects who did not eat either fish or meat on a regular basis were excluded. Subjects with NP or CWP were required to rate their pain >0 on a numeric rating scale (NRS) at the time of the blood sample collection. Subjects with a serum samples with visible haemolysis were excluded.

All subjects participated voluntarily after giving informed consent. The study was approved by the ethical committee of Uppsala University (registration number 2011/081) and carried out in accordance with relevant guidelines and regulations. All subjects were recruited consecutively over the same period (June 2011 until March 2012).

### General experimental procedure

The participants (all groups) were sent questionnaires to be answered and brought to the laboratory on the day of clinical examination. CON and subjects with NP were screened for inclusion/exclusion criteria by a trained physiotherapist. The NP group was also examined according to the protocols presented by Ohlsson *et al.* 1994^55^ with amendments Juul-Kristensen *et al.* 2006^56^ in order establish nonspecific neck-shoulder pain. Blood samples were collected within one week of the clinical investigation for the NP group and the CON.

CWP subjects were screened for inclusion/exclusion criteria by a medical doctor who specialised in rehabilitation medicine (SH), using a standardised protocol. All participants were asked not to use any pain medications except for paracetamol preparations for three days before the day of the experiment. Also, the subjects were asked to refrain from intake of caffeine, nicotine and wholegrains 12 h prior to the blood sample being collected and only to drink water in the last 2 h. All participants were asked about general diet habits, including if they eat a normal and varied diet, and detailed description of what they had eaten within 8 hours before the experiment. Participants were also asked not to perform any shoulder or neck-straining exercises 48 h before the study; they could do ordinary daily work and/or leisure activities. At the time of blood sample collection, all subjects rated their current pain and average pain during the last week, using a numerical rating scale (0–10).

### Blood sampling procedure

After subjects arrived at the laboratory they were allowed to rest for 15 min. Venous blood was drawn from the bend of the arm using the vacutainer system from BD Medical (Franklin Lakes, New Jersey, USA). All subjects were in a sitting position. The blood for serum analysis was treated according to a standardised protocol, which consisted of turning the vacutainer serum tube 10 times during 30 seconds at RT and then leaving the tube standing for 30 min at RT. The blood was then immediately centrifuged for 5 min at 2500 rpm and 4 °C. The supernatant was allocated in pre- labelled homoploymer tubes in aliquots of 500 μl and frozen in –80 °C until analysis.

### Processing of samples

Prior to extraction, the samples were allowed to thaw at room temperature and were then put on ice. Next 225 μl of the extraction buffer (methanol/water 9:1, with 11 IS (internal standard) each of the concentration 7 ng/μl) was added to 50 μl of the serum sample. The mixtures were vortexed for approximately 10 s and extracted in a bead mill (MM301 vibration mill, Retsch GmbH & Co. KG, Haan, Germany) at 30 Hz for 2 min. After 2 h at 4 °C on ice, samples were centrifuged at 14000 rpm for 10 min at 4 °C. A 100 μl aliquot was transferred to a GC vial and evaporated using a speedvac to dryness. Methoxymation with 30 μl methoxyamine solution in pyridine (15 μg/μl) was carried out at RT for 1 h, after whi h 30 μl of heptanes (with 0.5 μg of methyl stearate as injection IS) were added^57^,^58^.

### Chemicals

The chemicals used for sample preparation were all of analytical grade, except as otherwise stated. The following were purchased from Cambridge Isotope Laboratories, (Andover, MA, USA): stable isotope labelled IS compound 13C5-proline, 2H4-succinic acid, 13C5, 15 N-glutamic acid, 1,2,3-13C3 myristic acid, 2H7-cholesterol and 13C4 disodium alpha-ketoglutarate. The following were purchased from Campro (Veenendaal, The Netherlands): 13C12-sucrose, 13C4-palmitic acid and 2H4-butanediamine. 2HCl and 13C6-glucose from Aldrich (Steinheim, Germany) and 2H6-salicylic acid from Icon (Summit, NJ, USA). Stock solutions of the IS were prepared either in purified and deionised water (Milli-Q, Millipore, Billerica, MA, USA) or in methanol (J.T.Baker, Deventer, Holland) at the same concentration, 0.5 μg/μl. Metyl stearate was purchased from Sigma (St. Louis, MO, USA). N-Metyl-N-trimethylsilyltriflouroacetamide (MSTFA) with 1% trimethylchlorosilane (TMCS) and pyridine (silylation grade) were purchased from Pierce Chemical Co, Dallas,TX, USA). Heptane was purchased from Fisher Scientific (Loughborough, UK).

### Gas chromatography-time-of-flight mass spectrometry (GC-TOF-MS)

A 1 μl aliquot of derivatised sample was injected in splitless mode by an Agilent 7683 Series autosampler (Aligent, Atlanta, GA) into an Aligent 6980 GC equipped with a 10 m × 0.18 mm id fused-silica capillary column chemically bonded with 0.18 μm DB5-MS stationary phase (J&W Scientific, Folsom, CA). The injector temperature was 270 °C. The carrier gas was helium, set at a constant flow of 1 mL/min through the purge flow rate of 20 ml/min and an equilibrant time of 1 min. The column temperature was initially kept at 70 °C for 2 min and then increased from 70 °C to 320 °C at 30 °C/min, where it was kept for 2 min. The effluent from the column was introduced to the ion source in the Pegasus III TOF-MS (Leco Corp., St Joseph, MI). The transfer temperature was set at 250 °C and the ion source temperature at 200 °C. Ions were generated by a 70 eV electron beam at a current of 2.0 mA. Masses were acquired from *m/z* 50 to 800 at a rate of 30 spectra per second, and the acceleration voltage was turned on after a solvent delay of 165 s. Retention indexes were calculated from the retention times obtained from the injection of a homologous series of n-alkanes (C_12_–C_32_) for each batch. All samples were run in randomised order^58^.

### Hierarchical multivariate curve resolution

Analysing complex samples produces overlapping peaks in the GC-chromatograms and thus mixed mass spectra from the TOF-MS analysis. To solve this problem, a multivariate curve resolution method named hierarchical multivariate curve resolution (H-MCR) has been applied. In short, the H-MCR generates a matrix where all MD samples are described by a common set of variables, each representing one metabolite^57^. The data can then be subjected to multivariate data analysis as presented below. A recently implemented internal validation step in the H-MCR algorithm assures extraction of robust and reliable metabolite profiles^59^. Together GC-MS and H-MCR constitute a reproducible platform that allows for generation of unbiased GC-MS metabolomics for multiple sample comparisons within and between sample batches^57^,^59^.

### Univariate statistics

The analyses of the background variables and physical activity were conducted using non-parametric test since there was unequal distribution in the number of subject between the CWP group and the other two groups and the data was not normally distributed (Shapiro–Wilk test). To test for group difference between all three groups, Kruskal-Wallis tests were used. A Mann-Whitney-U test was used to test differences between the pain groups. Differences between the groups in self-rated leisure physical activity were analysed with Fisher’s exact test. The difference in individual metabolites between CON–NP and CON–CWP respectively were analysed by one-way ANOVA. A p-value <0.05 was used as the significance criterion in all analyses. The false discovery rate (FDR)^60^ was used to control for the expected proportion (5%) of incorrectly rejected null hypotheses (i.e. false discoveries) due to multiple comparisons in the one-way ANOVAs. FDR generates an adjusted significance criterion (q-value) for each test conducted. The original one-way ANOVA p-value should be less than the q-value for a correct rejection of the null hypothesis. Self-reported food intake 8 hour prior to blood sampling was analysed by categorising the components of the meals into two classes, meal containing 1) proteins, carbohydrate and fat; 2) meal lacking one or more components. In addition, the meals were classified according to if the subject reported eating nuts, whole grain, fresh vegetables or any other phytosterol rich food or not. The difference between groups in food categories were analysed with Fisher’s exact test.

### Multivariate statistics

In order to analyse for systematic differences in systemic metabolite content, the metabolite data were modelled and interpreted for the complex interactions using both unsupervised PCA and supervised OPLS-DA^61^. The SIMCA-P+ version 12 software was used for all multivariate analysis. In datasets from GC-TOF-MS of metabolites, the number of variables (metabolites) greatly exceeds the number of observation and the variables are often highly correlated. The multivariate projection methods, PCA and OPLS-DA, used in this study have the advantage of being able to deal with such data by summarising the systematic variation in a few so called latent variables built up by co-varying metabolites, i.e. correlated metabolite patterns. PCA displays the internal correlation structure in the data in a way that maximises the explained variation, thus grouping observations that share a similar variation structure independent of group. OPLS-DA separates the systematic variation among the metabolites that is directly related to group separation from variation not related, or orthogonal, to group separation.

In more detail, OPLS-DA is a supervised multivariate data projection method used to relate a set of predictor variables (X or metabolites in this study) to a response matrix (Y) that represents predefined sample classes (healthy, localised pain or chronic widespread pain). This in contrast to an unsupervised method (e.g. PCA) where the variation between the x-variables are modelled to find associations only within x without information of predefined groups or classes steering the model. The supervised method can thus be used to predict class identity and to extract specific features from among the predictor variables (metabolites) distinguishing between the predefined sample classes. OPLS operates by dividing the systematic variation in X into two parts: one part is the linearity related to Y, and thus can be used to predict Y (i.e. predictive components), and one part is uncorrelated (i.e. orthogonal components) to Y. In this process each variable in X is associated with a weight w* for each model component, which represents the variable’s covariation with Y in that component. Metabolites with value w* cross-validated 95% confidence intervals, not including 0 for the predictive component in models CON–NP and CON–CWP, were used to highlight significant metabolites or metabolite patterns within each model.

In this study, the internal correlation structure within each group (CON, NP or CWP) was first visualised by PCA to identify possible outliers (data not shown). Strong outliers, i.e., outliers that conforms to the overall correlation structure of the data but strongly changes the principal components, were identified by Hotelling T^2^ (with 95% CI) statistics in conjunction with the PCA-score plot for each group. Moderate outliers were identified by considering the residuals for each observation with a cutoff determined by p = 0.05 in an approximated F-distribution of the squared residuals of all observations. Then, one PCA model with all groups was constructed in order to visualise the internal correlation structure in x-space of the whole dataset (Fig. 1). Three OPLS-DA models with CON–NP-CWP (Fig. 2), CON–NP and CON–CWP were constructed to separate the systematic variation among the metabolites that is directly related to group separation. The shared and unique structures between the CON–NP and CON–CWP models were investigated using a SUS-plot (shared and unique structures). The SUS-plot consists of plotting the correlation coefficient of the predictive component p(corr) for both models against each other^62^.

## Additional Information

**How to cite this article**: Hadrévi, J. *et al.* Systemic differences in serum metabolome: a cross sectional comparison of women with localised and widespread pain and controls. *Sci. Rep.* **5**, 15925; doi: 10.1038/srep15925 (2015).

## Figures and Tables

**Table 1:** Subject characteristics.

	Control (n = 39)	Localised Pain (n = 30)	Chronic widespread pain (n = 16)	p-value
Age [years]	50.5 (25–65)	50.5 (26–64)	53.5 (41–64)	0.219^a^
Weight [kg]	64 (50–92)	64 (50–100)	67 (55–140)	0.886^a^
BMI [kg × m^2^]	23 (18–31)	24 (19–32)	24 (21–43)	0.510^a^
NRS-week	—	4 (1–8)	5,5 (2–7)	0.008*^,b^
NRS-day	—	3 (1–6)	3,5 (1–8)	0.109^b^
Pain duration [months]	—	25 (5–288)	204 (60–360)	0.0001*^,b^
Nicotine users [No.]	4 (10%)	5 (17%)	5 (31%)	0.102^c^

Data with median (range) for all variables and groups apart from the number of nicotine users, which is presented as the number (percent in group). Significant differences between groups are noted with *.

BMI = Body mass index. NRS = numerical rating scale 0–10. Statistical tests, p-value < 0.05 is considered significant. ^a^Kruskal-Wallis test, ^b^Mann-Whitney-U test, ^c^Fisher Exact test.

**Table 2:** Identified metabolite associated with NP in the CON–NP OPLS-DA model.

Identified metabolite	w* [CI]	p-value	q-value^a^
Nonanoic acid	0.156 [0.127–0.186]	8.14 × 10^−18^*	0.0006^#^
D-Glucose	0.117 [0.091–0.143]	3.65 × 10^−8^*	0.0041^#^
D-Glucose	0.114 [0.079–0.148]	3.86 × 10^−7^*	0.0058^#^
D-Glucose	0.104 [0.079–0.128]	4.41 × 10^−7^*	0.0056^#^
Adenosine-5-monophasphate	0.098 [0.045–0.152]	6.24 × 10^−5^*	0.0095^#^
Tetradecanoic acid (Myristic acid)	0.088 [0.062–0.115]	0.0006*	0.0134^#^
cis-11- Eiocosenoic acid (Gondoic acid)	0.083 [0.03–0.136]	0.0001*	0.0103^#^
Inositol-1-phosphate	0.082 [0.025–0.138]	0.0016*	0.0148^#^
Succininc acid	0.077 [0.043–0.111]	0.0008*	0.0136^#^
Octadecadienoic acid (Linoleic acid)	0.062 [0.014–0.11]	0.0023*	0.0167^#^
Saccharopine	0.051 [0.008–0.094]	0.047*	0.0257
Hexadecanoic acid (Palmitic acid)	0.05 [0.004–0.096]	0.019*	0.0222^#^
Octadec-9-enoic acid (Oleic acid)	0.05 [0–0.099]	0.018*	0.022^#^
O-phosphoethanolamine	0.043 [0.006–0.079]	0.211	0.0337
L-Valine	0.042 [0.005–0.079]	0.031*	0.0245
Arachidonic acid	0.038 [0.007–0.069]	0.033*	0.0247
L-Aspartic acid	0.034 [0.001–0.066]	0.027*	0.0243

Metabolites have w*cross-validated confidence intervals (95%) not including 0. * = significant change from CON p>0.05 (one-way ANOVA). ^#^ = significant change from CON (one-way ANOVA) after false discovery rate correction^60^. Metabolites are sorted with the most positive w* on top, i.e. the most closely associated with NP in the OPLS-DA model. Multiple entries of the same metabolite reflect different degrees of derivatisation or split peaks.

^a^The q-value for each individual ANOVA is the minimal level at which the p-value of that ANOVA may be considered significant^60^.

**Table 3:** Identified metabolite associated with CON in the CON–NP OPLS-DA model.

Identified metabolite	w* [CI]	p-value	q-value^a^
Threonic acid	−0.137 [−0.176– −0.098]	1.2 × 10^−8^*	0.0035^#^
DL-Cysteine	−0.136 [−0.177– −0.096]	2.7 × 10^−9^*	0.0033^#^
Erythrose-4-phosphate	−0.125 [−0.149– −0.101]	1.3 × 10^−7^*	0.0047^#^
Cholesterol	−0.119 [−0.157– −0.081]	4.8 × 10^−7^*	0.006^#^
Inosine	−0.117 [−0.151– −0.083]	8.9 × 10^−8^*	0.0045^#^
DL-Glycerol-3-phosphate	−0.112 [−0.151– −0.073]	4.8 × 10^−6^*	0.007^#^
Cholesterol	−0.104 [−0.145– −0.062]	2.1 × 10^−5^*	0.0086^#^
DL-Glutamine	−0.094 [−0.127– −0.061]	4.3 × 10^−6^*	0.0066^#^
L-Serine	−0.093 [−0.124– −0.062]	0.0001*	0.0105^#^
Octadecanoic acid (Stearic acid)	−0.085 [−0.122– −0.049]	0.0023*	0.0154^#^
Creatinine	−0.081 [−0.118– −0.044]	0.0029*	0.0165^#^
DL-Serine	−0.077 [−0.114– −0.04]	0.0024*	0.0160^#^
Taurine	−0.074 [−0.111– −0.037]	0.0022*	0.0152#
DL-Glyceric acid	−0.07 [−0.108– −0.033]	1.1 × 10^−5^*	0.0076^#^
Asparagine	−0.07 [−0.109– −0.03]	0.0031*	0.0171^#^
Arginine	−0.069 [−0.099– −0.038]	0.0033*	0.0177^#^
α-Tocopherol (Vitamin E)	−0.067 [−0.093– −0.041]	0.0054*	0.0185^#^
Campesterol	−0.064 [−0.106– −0.021]	0.0173*	0.0216^#^
Monomethylphosphate	−0.062 [−0.104– −0.019]	0.0150*	0.0212^#^
Ornithine	−0.061 [−0.086– −0.035]	0.0170*	0.0214^#^
L-Tyrosine	−0.06 [−0.099– −0.021]	0.0107*	0.0202^#^
D-Mannose	−0.059 [−0.091– −0.028]	0.0073*	0.0189^#^
L-Cystine	−0.058 [−0.103– −0.012]	0.0241*	0.0235
Aminomalonic acid	−0.055 [−0.073– −0.037]	0.0014*	0.0142^#^
D(-)Quinic acid	−0.055 [−0.088– −0.021]	0.0533	0.0259
Pipecolic acid	−0.052 [−0.098– −0.007]	0.0540	0.0261
β—Sitosterol	−0.049 [−0.083– −0.015]	0.0831	0.0275
Benzenebutanoic acid	−0.044 [−0.082– −0.006]	0.0940	0.0276

Metabolites have w*cross-validated confidence intervals (95%) not including 0. * = significant change from NP p > 0.05 (one-way ANOVA). ^#^ = significant change (one-way ANOVA) from NP after false discovery rate correction^60^ i.e. have a p-value < q-value. Metabolites are sorted with the most negative w* on top, i.e. the most closely associated with CON in the OPLS-DA model. Multiple entries of the same metabolite reflect different degrees of derivatisation or split peaks.

^a^The q-value for each individual ANOVA is the minimal level at which the p-value of that ANOVA may be considered significant^60^.

**Table 4:** Identified metabolite associated with CWP in the CON–CWP OPLS-DA model.

Identified metabolite	w* [CI]	p-value	q-value^a^
Saccharopine	0.111 [0.063–0.159]	0.0793	0.0049
Chenodeoxycholic acid	0.106 [0.059–0.152]	0.1105	0.0066
L-Cystine	0.097 [0.015–0.18]	0.0951	0.0062
Xylose or Ribose	0.066 [0.006–0.127]	0.2716	0.0142

Metabolites have w*cross-validated confidence intervals (95%) not including 0. * = significant change from CON p > 0.05 (one-way ANOVA). No metabolites are significantly (one-way ANOVA) different between after false discovery rate correction, i.e. have a p-value < q-value. Metabolites are sorted with the most positive w* on top, i.e. the most closely associated with CWP in the OPLS-DA model.

^a^The q-value for each individual ANOVA is the minimal level at which the p-value of that ANOVA may be considered significant^60^.

**Table 5:** Identified metabolite associated with CON in the CON–CWP OPLS-DA model.

Identified metabolite	w* [CI]	p-value	q-value
Threonic acid	−0.185 [−0.259– −0.11]	0.0009*	0.0002
Cholesterol	−0.099 [−0.194– −0.003]	0.0898	0.0058
Campesterol	−0.092 [−0.134– −0.05]	0.1165	0.0070
D(-)Quinic acid	−0.082 [−0.138– −0.026]	0.1469	0.0084
β-Sitosterol	−0.053 [−0.098– −0.008]	0.3672	0.0191

Metabolites have w*cross-validated confidence intervals (95%) not including 0. * = Significant change from CWP p > 0.05 (one-way ANOVA). No metabolites are significantly (one-way ANOVA) different between after false discovery rate correction i.e. have a p-value < q-value. Metabolites are sorted with the most negative w* on top, i.e. the most closely associated with CON in the OPLS-DA model.

^a^The q-value for each individual ANOVA is the minimal level at which the p-value of that ANOVA may be considered significant^60^.
